# Season, prey availability, sex, and age explain prey size selection in a large solitary carnivore

**DOI:** 10.1002/ece3.11080

**Published:** 2024-03-07

**Authors:** Logan Bates‐Mundell, Sara H. Williams, Kim Sager‐Fradkin, Heiko U. Wittmer, Maximilian L. Allen, Bogdan Cristescu, Christopher C. Wilmers, L. Mark Elbroch

**Affiliations:** ^1^ Faculty of Environment and Natural Resources University of Freiburg Freiburg im Breisgau Germany; ^2^ Panthera New York City New York USA; ^3^ Lower Elwha Klallam Tribe Natural Resources Port Angeles Washington USA; ^4^ School of Biological Sciences Victoria University of Wellington Wellington New Zealand; ^5^ Illinois Natural History Survey, Prairie Research Institute University of Illinois Champaign Illinois USA; ^6^ Environmental Studies Department University of California Santa Cruz California USA

**Keywords:** intraspecific variation, optimal foraging, prey selection, *Puma concolor*, wildlife management

## Abstract

Prey selection is a fundamental aspect of ecology that drives evolution and community structure, yet the impact of intraspecific variation on the selection for prey size remains largely unaccounted for in ecological theory. Here, we explored puma (*Puma concolor*) prey selection across six study sites in North and South America. Our results highlighted the strong influence of season and prey availability on puma prey selection, and the smaller influence of puma age. Pumas in all sites selected smaller prey in warmer seasons following the ungulate birth pulse. Our top models included interaction terms between sex and age, suggesting that males more than females select larger prey as they age, which may reflect experiential learning. When accounting for variable sampling across pumas in our six sites, male and female pumas killed prey of equivalent size, even though males are larger than females, challenging assumptions about this species. Nevertheless, pumas in different study sites selected prey of different sizes, emphasizing that the optimal prey size for pumas is likely context‐dependent and affected by prey availability. The mean prey weight across all sites averaged 1.18 times mean puma weight, which was less than predicted as the optimal prey size by energetics and ecological theory (optimal prey = 1.45 puma weight). Our results help refine our understanding of optimal prey for pumas and other solitary carnivores, as well as corroborate recent research emphasizing that carnivore prey selection is impacted not just by energetics but by the effects of diverse ecology.

## Graphical abstract

Season, prey availability, and age influence the size of prey selected by pumas across six sites in North and South America. The optimal prey size for pumas appears context‐dependent, although evidence suggests it is smaller than predicted by ecological theory. Males and females killed similar‐sized prey, even though males are much larger, and males more than females killed larger prey as they aged.

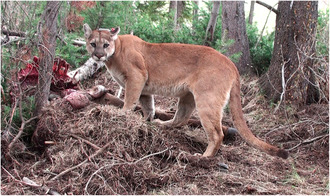

## 1 INTRODUCTION

Prey selection and predator–prey dynamics are fundamental aspects of ecology that drive evolution and community structure, yet the impact of intraspecific variation in these processes, although acknowledged, remains largely unaccounted for in ecological theory (Bump et al., 2022; Chesson, 1978; Pettorelli et al., 2015). Individual variation in prey selection appears to be driven by multiple intrinsic and extrinsic factors, including life history, intraspecific competition, age and behavioral stage, and the diversity of available resources. For example, sexual dimorphism in some species has been shown to influence prey selection (White et al., 2011). Prey selection is also largely dependent upon prey availability and prey vulnerability, which varies with season and across ecosystems (Allen et al., 2014; Clark et al., 2014; Day et al., 2015; Metz et al., 2012). Further, the suite of potential drivers that act on any one population vary in space and time (Nakayama et al., 2017; Newsome et al., 2015; Pettorrelli et al. 2011).

Individual prey selection among solitary carnivores likely impacts predator–prey dynamics in multi‐prey systems (e.g., prey switching, Vettorazzi et al., 2022), such as the population viability of rare prey (Festa‐Bianchet et al., 2006; Ross et al., 1997; Wittmer et al., 2014). It may also provide insights into the ecology of different life stages of a species (Elbroch, Feltner, & Quigley, 2017a), as well as social tolerance for (Treves & Karanth, 2003) and management of (Linnell et al., 1999) carnivores in general. For example, individual pumas (*Puma concolor*), rather than entire populations, tend to select for rare prey such as huemul deer (*Hippocamelus bisulcus*) and bighorn sheep (*Ovis canadensis*) in multi‐prey systems (Festa‐Bianchet et al., 2006; Wittmer et al., 2014), and therefore, effective management needs to address selection by individuals rather than populations (Graham et al., 2011). Including the stochastic presence of rare prey specialists also extends the estimated viability of rare prey species (Wittmer et al., 2014), reducing the relative severity of the effects of predation on rare prey species. Individual pumas and other carnivores that target ecosystem engineers and other keystone species, such as porcupines (*Erethizon dorsatum*) and American beavers (*Castor canadensis*), may also have a disproportionate impact on ecosystem function and structure (Bump et al., 2022; Gable et al., 2020; LaBarge et al., 2022; Lowrey et al., 2016). Young bobcats (*Lynx rufus*) (Litvaitis et al., 1986), cheetahs (*Acinonyx jubatus*) (Caro, 1994), and pumas (Elbroch, Feltner, & Quigley, 2017a) all exhibit selection for smaller prey, especially during dispersal, and this variation supports a critical stage of life essential to maintaining genetic connectivity within a metapopulation structure (Sweanor et al., 2000). However, these topics have yet to receive much research attention.

Pumas are wide‐ranging apex carnivores that play crucial roles in supporting biodiversity and ecosystem resilience through direct predation, interactions with other carnivores and scavengers, and through their influence on prey spatial distributions and behaviors (LaBarge et al., 2022; Laundré, 2010). That pumas exhibit individual variation in foraging at the site level is well‐established (e.g., Elbroch et al., 2016; Lowrey et al., 2016; Ross et al., 1997); however, we have yet to assess whether there are patterns of intraspecific prey selection that hold true across study systems.

We tested three hypotheses regarding prey selection in pumas across six study sites. First, pumas eat larger prey under three conditions: (1) in winter, (2) in sites where larger prey are available (e.g., systems with vs. without elk, *Cervus canadensis*), and (3) with increasing age. Second, we hypothesized that males, which are larger than females in this species, select larger prey than females. Third, pumas select prey 1.45 times larger than mean puma weight, which is predicted to be their optimal prey size based on energetic modeling (Carbone et al., 1999). Every carnivore is expected to have an “optimal” prey size (Elton, 1927), which they select for and attack more often than other prey sizes (Brose, 2010).

## 2 MATERIALS AND METHODS

### 2.1 Study sites

**FIGURE 1:**
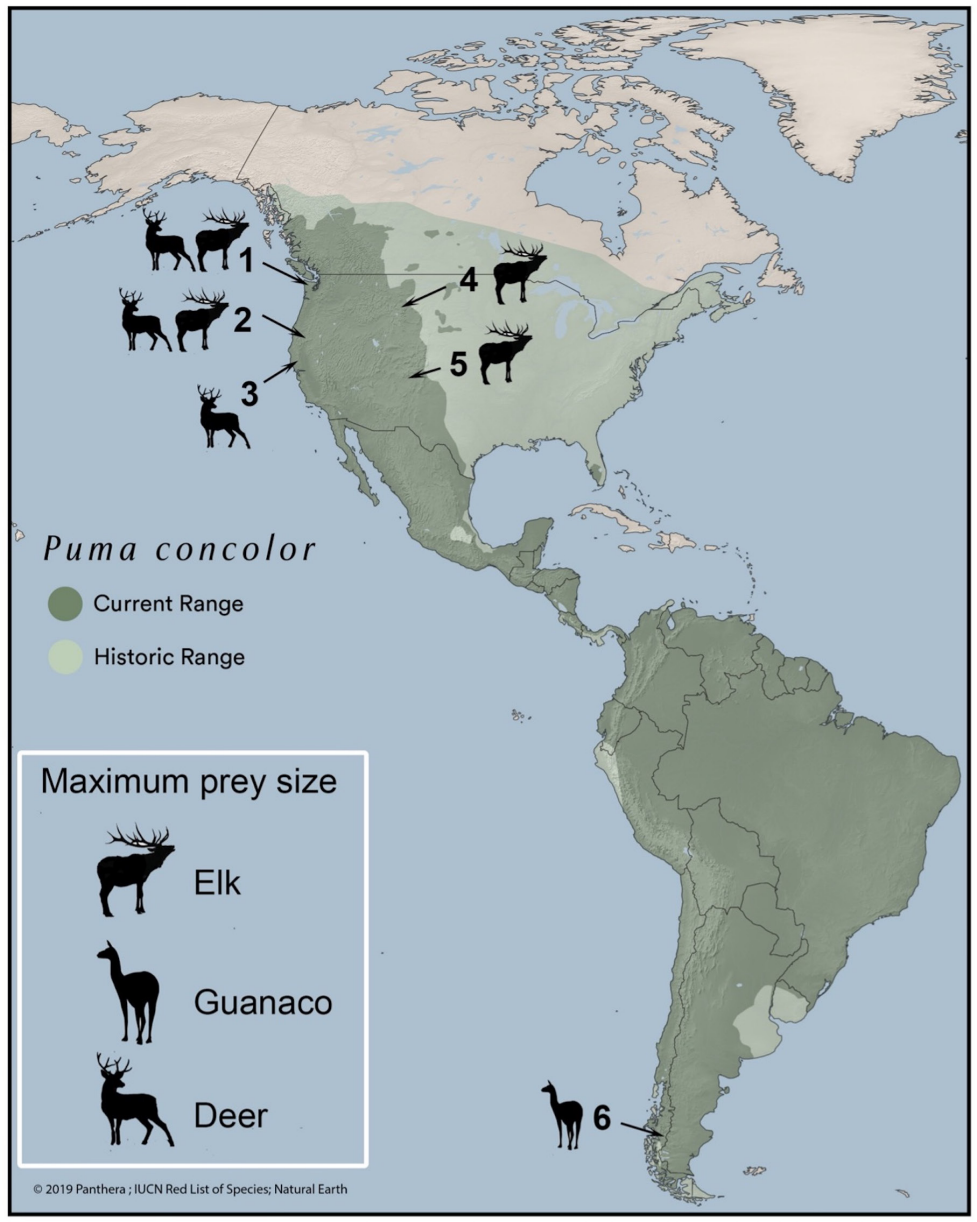
Puma range, the location of our six study sites and the maximum prey sizes in each site. 1—Olympic Peninsula, 2—Siskiyou, 3—Mendocino, 4—Wyoming, 5—Colorado, 6—Patagonia.

We conducted research across six study sites in North and South America (listed alphabetically below), where pumas were followed intensively for the duration the animal wore a functional collar or for long blocks of time for focal sampling, so as to ensure equal probabilities of sampling kills of different size (see Elbroch et al. (2018) for a discussion of determining prey selection via modeling versus intensive fieldwork) (Figure 1). For each study site description, we also include the large terrestrial competitors for pumas.

#### 2.1.1 California, USA—Mendocino County

The Mendocino study site was located within the Mendocino National Forest and adjacent private lands in northern California, USA (W 39.738, S −123.160). Pumas in this system predominantly preyed upon black‐tailed deer (*Odocoileus hemionus columbianus*), but also fed on California ground squirrels (*Otospermophilus beecheyi*), Western gray squirrels (*Sciurus griseus*), and black‐tailed jackrabbits (*Lepus californicus*). The largest available prey was black‐tailed deer. Puma competitors in Mendocino predominantly consisted of American black bears (*Ursus americanus*) (Allen et al., 2021). Additional details regarding topography, precipitation, and plant and animal communities for this site are found in Allen et al. (2014).

#### 2.1.2 California, USA—Siskiyou County

The Siskiyou study site was located in Northern California near the town of Mount Shasta (N 41.310°, W −122.311°). Ungulate prey available within this site included mule deer (*O. hemionus*), Roosevelt elk (*C. c. roosevelti*), and pronghorn (*Antilocapra americana*), as well as a population of feral horses (*Equus caballus*). The largest available prey was elk or horse, although we did not detect any predation of feral horses. Puma competitors in Siskiyou predominantly consisted of American black bears. Additional details regarding topography, precipitation, and plant and animal communities for this site are found in Wittmer et al. (2021).

#### 2.1.3 Colorado, USA—Garfield County

The Colorado study site was located near the town of De Beque, Colorado, USA (W 39.385°, S −108.324°). Puma prey included Rocky Mountain elk (*C. c. nelsoni*), mule deer, and small numbers of moose (*Alces alces*). The largest available prey for individual pumas was either elk or moose. Puma competitors in Colorado included American black bear. Additional details about topography, precipitation, and plant and animal communities are found in Elbroch et al. (2014).

#### 2.1.4 Patagonia, Chile—Patagonia National Park

The Patagonia site was located in Patagonia National Park in the southern Aysén Region of Chilean Patagonia (W 47.800°, S 72.000°). Ungulate prey included guanacos (*Lama guanicoe*), huemul deer (*Hippocamelus bisulcus*), and domestic sheep (*Ovis aries*). The largest available prey was the guanaco. Pumas were the apex carnivore in the system and lacked large terrestrial competitors. Additional details about topography, precipitation, and plant and animal communities are found in Elbroch and Wittmer (2012).

#### 2.1.5 Washington, USA—Olympic Peninsula

The Olympic site was located on the northwest Olympic Peninsula in Clallam County, Washington, USA (N 48.112°, W −123.776°). Local ungulate species included Roosevelt elk, and black‐tailed deer. The largest available prey varied among pumas, as only a subset of pumas had access to elk in addition to deer. Puma competitors consisted of American black bears. Additional details regarding topography, precipitation, and plant and animal communities for this site are found in McCaffery et al. (2020) and Stratton et al. (2022).

#### 2.1.6 Wyoming, USA—Greater Yellowstone Ecosystem

The Wyoming site was in the southern Greater Yellowstone Ecosystem (N 43.680°, W −110.270°). Ungulate prey included bighorn sheep, Rocky Mountain elk, moose, mule deer, pronghorn, and a small population of white‐tailed deer (*O. virginianus*). The largest available prey for individual pumas was elk or moose. Puma competitors included gray wolves (*Canis lupus*), grizzly bears (*U. arctos*), and American black bears. Additional details about topography, precipitation, and plant and animal communities are found in Elbroch et al. (2013).

### 2.2 Puma captures, ethics, and aging

Research teams accomplished puma captures using trained scent‐trailing dogs (with the assistance of dog handlers), box traps, and foot snares (see Elbroch et al., 2013 and Elbroch et al., 2014 for details about capture protocols). All puma capture and handling protocols followed guidelines developed by the American Society of Mammologists (Sikes & Gannon, 2011) and were approved by independent Institutional Animal Care and Use Committees (University of California Davis Protocols 13252, 15341, 16645, 16886; University of California, Santa Cruz, Protocol number Wilmc1101; University of Idaho Protocol IACUC‐2020‐15, Jackson Protocol 027‐10EGDBS‐060210; National Park Service IACUC Protocol IMR_GRTE_Elbroch_Cougar_2013–2015).

#### 2.2.1 GPS programming and identifying puma prey

We programmed GPS collars to obtain location data at 1‐ or 2‐h intervals (i.e., 12 or 24 locations/day). GPS data were transmitted through an Argos uplink at 3‐day intervals in Patagonia and Mendocino, or 2–6 times per day via Iridium and Globalstar uplinks for the remaining sites.

We identified aggregated GPS points, termed GPS clusters (Anderson Jr & Lindzey, 2003), via visual assessments in GoogleEarth or ArcGIS, except in Siskiyou and Washington, where we employed a Python script (Python Software Foundation Hampton, NH) to assess GPS data and identify clusters. We defined clusters as any ≥2 points within 150 m of each other spanning 2 h to 2 weeks, except in Wyoming and Washington, where we identified clusters that spanned 4 h to 2 weeks, and Mendocino, where identified clusters spanned 8 h to 2 weeks. Researchers investigated GPS clusters in the field using handheld GPS units to navigate to sites, and assessed hair, skin, rumen, and bone fragments to identify prey species and sex. We differentiated predation from scavenging based upon associated signs, including bite marks, blood splatter, and signs of chase or struggle (Elbroch et al., 2013). Ungulate prey age was determined based on tooth eruption sequences and lower mandible wear, with individuals ≥3 years considered as adults (Elbroch et al., 2013). We determined prey weights from the published literature and, in some cases, utilized ungulate neonate growth curves (Tables A1 and A2 in Appendix).

### 2.3 Statistical analyses

We evaluated 10 a priori candidate models (Table 1) that tested varying aspects of our three hypotheses in R Statistical Software (Version 4.2.2, R Core Team, 2022). To determine whether pumas killed larger prey in winter, in sites where larger prey were available, and with increased age (our first hypothesis), we utilized the fixed effect variables *season*, *site* (i.e., research site), *max prey* (prey availability) and puma *age*. We examined the prediction that males will select larger prey than females (our second hypothesis) using variable *sex* and interaction terms sex*age, as well as sex*max prey. To test our third hypothesis, we calculated *mean prey size* that pumas utilized at both the site and the multi‐site level.

**TABLE 1:** Ranked models of prey weight used by pumas based on Akaike Information Criterion corrected for small sample sizes (AICc) scores and weights.

	Models	AICc	ΔAICc	Likelihood	w_*i*_	*k*
Model 1	age*sex + max prey + season + (1 | ID)	33,934.63	0.00	1.00	0.38	11
Model 2	max prey + season + (1 | ID)	33,935.48	0.85	0.65	0.25	8
Model 3	age + max prey + season + (1 | ID)	33,936.06	1.43	0.49	0.19	9
Model 4	age*sex + max prey*sex + season + (1 | ID)	33,937.01	2.38	0.30	0.12	13
Model 5	max prey*sex + season + (1 | ID)	33,938.89	4.26	0.12	0.045	11
Model 6	max prey*sex + age + season + (1 | ID)	33,939.86	5.23	0.07	0.027	12
Model 7	age*sex + season + (1 | ID)	33,950.74	16.11	0.00	0.00	9
Model 8	age*sex + max prey + (1 | ID)	34,082.53	147.90	7.65E‐33	2.90E‐33	8
Model 9	age*sex + max prey*sex + (1 | ID)	34,084.26	149.63	3.22E‐33	1.22E‐33	10
Model 10	age*sex + (1 | ID)	34,097.61	162.98	4.07E‐36	1.54E‐36	6

*Note*: Model variables include age (puma age at time of kill), puma sex, max prey (maximum size prey available at site), season, and ID (random effect puma ID). Model descriptions, including variables, are followed by the AICc score, the change in AICc values (∆AICc), the model likelihood, Akaike weights (w*i*) and number of parameters (*k*).

We determined seasonal classifications (*season*) based on ungulate parturition dates at each site, which occur in late May and early June for ungulates, including deer and elk across California, Wyoming, Washington, and Colorado (Bowyer, 1991; Hines & Lemos, 1979; Peterson et al., 2017; Smith, 1994; Whittaker & Lindzey, 1999), and November and December in Patagonia (Corti et al., 2010; Gonzalez et al., 2006). For northern sites, we defined summer as the 3 months from May 15 to August 15, and then Autumn, Winter, and Spring as the 3‐month intervals following summer. In Patagonia, we defined summer as the 3‐month interval from November 15 to February 15, and then Autumn, Winter and Spring following at 3‐month intervals.

We categorized the largest prey available to each puma in its home range (*max prey*) using a categorical variable that was based on prey weight (3 values: deer, guanaco, elk). We classified puma *age* (months) using gum line recession measured at captures, following Laundré et al. (2000), or by birthdate for pumas for which we knew this information. We estimated puma age at the time of each kill by adding an individual's age at capture to the number of days since said capture before the kill was made. We log‐transformed *age* at the time of the kill for analyses. We determined puma *sex* (M or F) at the capture event.

We used Generalized Linear Models (GLMs) with a log‐link function and gamma distribution for hypothesis testing. In our gamma regression analyses, we used *prey weight* (in kg) as the response variable. To estimate *prey weight* for each prey item that pumas consumed at a site, we excluded prey with neither discernible age nor sex characteristics. We assigned prey with identifiable age characteristics but no discernible sex the median species‐specific weight for males and females within that age class. We excluded kill sites with no corresponding date for the kill from this analysis.

We included a random effect for puma (*ID*) to avoid pseudoreplication and biases introduced by sampling one puma more than another. We used variance inflation factors (VIF) to assess multicollinearity among covariates. Among correlated covariates, we considered any VIF scores >2 to have large impacts, with VIF >5 considered highly correlated and VIF >10 considered a severe correlation (Graham, 2003). We fit all 10 models using the “lme4” package (Bates et al., 2015) in R. We ranked models using Akaike's Information Criterion corrected for small sample size (AICc) using “AICcmodavg” package (Mazerolle, 2023) in R. We considered any model within ∆AICc <2 of the lowest AICc model as top models (Burnham & Anderson, 2002).

We conducted post hoc ANOVA tests to determine whether pumas selected different prey sizes at different sites. When a significant *p*‐value was generated, we assumed at least two sites had significant differences. To investigate this further, we ran a Tukey's HSD test for site comparisons.

Finally, we calculated mean prey size for pumas as compared to mean puma weights, to test the assumption that mean prey size would be 1.45 times larger than mean puma weight, following Carbone et al. (1999) optimal prey size estimates.

## 3 RESULTS

We collected data across our six study sites from 81 independent pumas (41 females and 40 males). Their weight ranged from 32 to 82 kg. In total, we collected data from 3325 individual kill sites (X̄ = 41.05 ± 46.48 SD kills per puma, X̄ = 54.34 ± 54.29 SD kills per female, X̄ = 27.43 ± 32.30 SD kills per male) representing 85 species of prey, including 12 species of ungulates. The youngest independent puma was 10 months (Olympic site) and the oldest was 146 months (Wyoming site).

### 3.1 Foraging strategies

The global model that included all six covariates (*age*, *ID*, *sex*, *season*, *site*, and *max prey*) failed to converge. Variance inflation factors indicated that *site* (VIF = 5.597) and *max prey* (VIF = 4.174) were highly correlated. Therefore, we removed the covariate “*site*” from further analyses.

All remaining candidate models used a random effect of the individual puma (to account for repeat measures from individuals) and different subsets of the following variables: age, sex, maximum prey size, season, as well as two interaction terms: one for the interaction between age and sex, and the other for the interaction between maximum prey available and sex (Table A3 in Appendix). Of the 10 models that we ran, we identified three top models, with Model 1 receiving the most support (Table 1).

**FIGURE 2:**
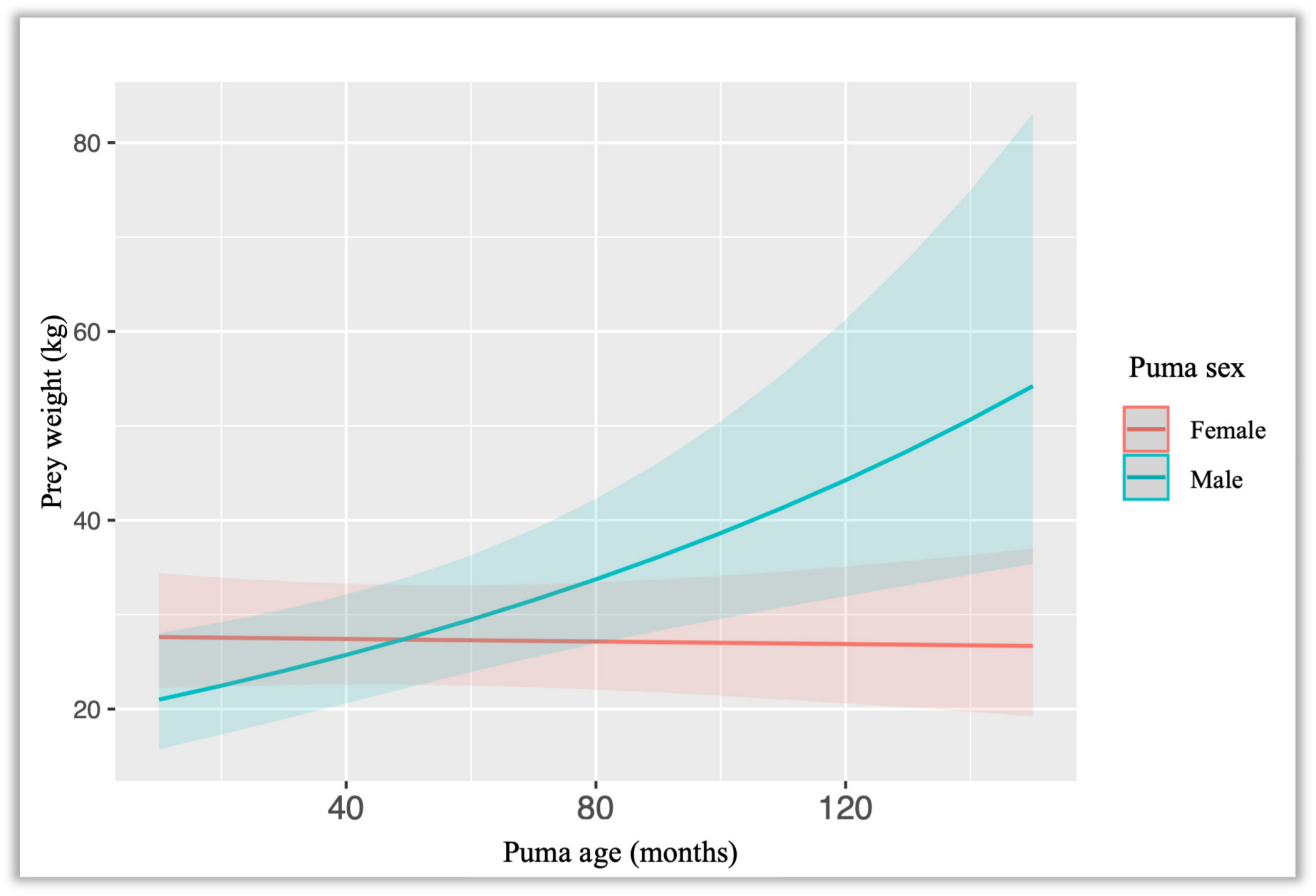
Interaction effect for Model 2, illustrating the interaction of puma age and puma sex.

Based on Model 1, pumas selected larger prey in spring (*β* = 0.381 *±* 0.051, *p* < .001) and winter (*β* = 0.416 *±* 0.053, *p* < .001) as compared to autumn, and in sites where larger prey (elk) were available (*β* = 0.598 *±* 0.13, *p* < .001) as compared to sites with only deer. In addition, age had a significant interaction with sex (*β* = 0.207 *±* 0.090, *p* = .022), such that increasing age had a positive influence on prey size for males (Table A3 in Appendix) but did not have a significant influence on prey size for females (Figure 2). Model 2 was similar to Model 1, with pumas selecting larger prey in spring (β = 0.371 *±* 0.050, *p* < .001) and winter (β = 0.411 *±* 0.053, *p* < .001) as compared to autumn, and in sites where larger prey (elk) were available (β = 0.545 *±* 0.13, *p* < .001) as compared to sites with only deer. Based on Model 3, pumas selected larger prey in spring (β = 0.376 *±* 0.051, *p* < .001) and winter (β = 0.414 *±* 0.053, *p* < .001) as compared to autumn, and in sites where larger prey (elk) were available (β = 0.547 *±* 0.12, *p* < .001) compared to deer only sites. Model 3 also included puma age, but the confidence intervals did not overlap zero for this parameter estimate.

### 3.2 Sex‐biased selection and site‐specific average prey densities

**FIGURE 3:**
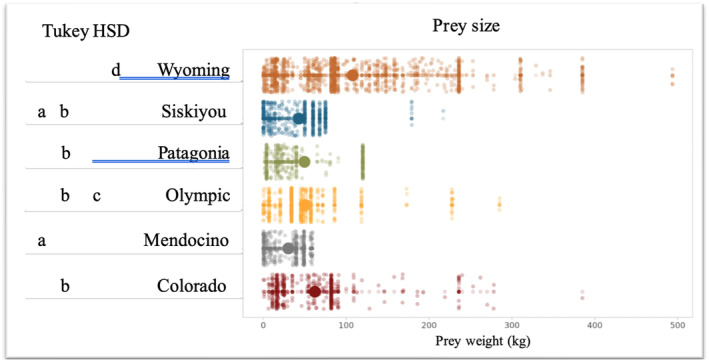
Average prey size and prey size ranges utilized by pumas across sites (right). Results of a post hoc Tukey's HSD test which differentiated prey size among sites (left). Sites with the same letter had statistically equivalent prey size use by pumas.

Pumas in our study weighed 49.3 kg *±*12.7 SD, resulting in predicted prey weights of 71.4 kg *±*18.5 SD. Weights of prey that pumas used were equivalent to predicted prey weights as described by Carbone et al. (1999), but only because of the large variation in prey selected by pumas, resulting in very large SDs for prey weight. Ignoring variable sampling of individuals, the mean of all kill weights was 68.3 kg and the median was 50.0 kg. When accounting for differential sampling of individual pumas, male (56.7 kg *±*5.2 SD) and female (60.3 kg *±*5.5 SD) pumas killed prey of equivalent size (*F*_1,76_ = 0.23, *p* = .633). Pumas in different sites, however, selected different sized prey (Figure 3). Pumas in Mendocino and Siskiyou selected the smallest average prey, and pumas in Wyoming selected the largest. The results of our Tukey's HSD comparisons are found in Figure 3.

## 4 DISCUSSION

Our results highlight the strong influence of season and prey availability on puma prey size selection across sites representing diverse ecological variation, which have been emphasized in recent literature (Allen et al., 2014; Cristescu et al., 2022; Knopff et al., 2010), and the smaller influence of puma age on prey selection (Elbroch & Quigley, 2019). Across six sites, male and female pumas killed relatively similarly sized prey, challenging assumptions that larger males characteristically kill larger prey than females. Mean puma prey weight across all sites was 1.18 times mean puma weight rather than the predicted 1.45 times, and the median prey weight was even smaller. Nevertheless, the range of prey weights selected by pumas fell within the predicted range proposed by Carbone et al. (1999). Our findings support recent research on puma foraging that emphasizes their selection not for larger, heavier adult ungulates, but for smaller, younger ones, that are likely more vulnerable to attack (e.g., Elbroch, Feltner, & Quigley, 2017a). Research on puma caching also highlights the importance of intermediate‐sized prey, which they cache more often than larger or smaller prey, likely to increase foraging time and thus energetic value (Allen et al., 2023).

We found substantial support for our first hypothesis, which predicted that pumas would eat larger prey in winter, in sites where larger prey were available, and with increased age. Trends demonstrated throughout the data showed an interaction of variables influencing puma foraging strategies across our six study sites including season, prey availability, and age of individual puma. Pumas generally selected smaller prey in summer and fall in temperate regions, following ungulate birth pulses and the seasonal availability of smaller, more vulnerable neonates and other prey (Knopff et al., 2010). Pumas may also have selected smaller prey during these warmer months due to increased presence of bears (Elbroch et al., 2015) as well as greater decomposer activity (Allen et al., 2014). Our results also provided additional support for the idea that pumas select larger prey if available, especially male pumas (Elbroch et al., 2013; Knopff et al., 2010; White et al., 2011). Further, puma age appeared in two of the three top models and puma sex in one of the top models. The overall trend showed that when accounting for interactions between puma age and sex, older pumas were associated with the selection of heavier prey, especially males (e.g., Elbroch et al., 2013; Knopff et al., 2010; White et al., 2011).

We found little support for our second hypothesis that males would select larger prey than females. When we assessed prey weight more directly, mean prey size for females was statistically equivalent to that of males. Other studies have reported that male pumas select larger prey than females (Clark et al., 2014; Elbroch et al., 2013; Knopff et al., 2010; White et al., 2011), but our results suggest that although males do occasionally kill very large prey where available, this may not be common puma behavior. Prey size also impacts kill rates, assuming that kill rates reflect energetic requirements (Brose, 2010), and although there is evidence that male pumas, which sometimes weigh twice as much as adult females, exhibit lower kill rates than females with dependent young, they exhibit similar rates to females without young (Cristescu et al., 2022). We hypothesize that males meet their larger energetic requirements not necessarily by killing larger prey or by killing more frequently, but instead by eating more of each prey item they kill (Elbroch et al., 2014) or scavenging from kills of females as part of social networks (Elbroch, Levy, et al., 2017).

We found weak support for our third hypothesis regarding the optimal prey size for pumas, which we could interpret as evidence that this was an inappropriate question across diverse landscapes with different‐sized prey. Nevertheless, our results did indicate that pumas appear to kill smaller prey than predicted by energetic models, contributing to a growing body of evidence that is redefining our understanding of the foraging behavior of this species, and potentially other solitary felids as well. Recent research suggests that selecting smaller prey is driven by diverse ecology, including mitigating competition with other carnivores (e.g., American black bears, Allen et al., 2021; gray wolves, Kortello et al., 2007), the likeliness of kleptoparasitism and an energetic balance between prey size and satiation (Allen et al., 2023), or alternatively, social learning and life stage (Elbroch, Feltner, & Quigley, 2017a, 2017b; Elbroch, Levy, et al., 2017; Elbroch & Quigley, 2019).

There is still considerable work needed to understand when and why pumas select for smaller prey, given its potential influence on diverse ecology ranging from seasonal prey vulnerability (Knopff et al., 2010) to the potential impacts of competitors (Allen et al., 2021). We also encourage further research on the role of small prey in maintaining healthy puma and other large carnivore populations, especially for dispersing animals vital to connecting populations within a metapopulation framework (Sweanor et al., 2000). Research on felids has shown that younger, less experienced animals sometimes select smaller prey. Examples include bobcats (Litvaitis et al., 1986), house cats (*Felis catus*, Kitchener, 1999), cheetahs (Caro, 1994), African lions (*Panthera leo*; Hayward et al., 2007), and pumas (Elbroch, Feltner, & Quigley, 2017a). This selection by young felids of smaller prey reflects learning the skills of hunting and handling prey. Alternatively, selection for smaller prey may reflect a lack of familiarity with the large prey landscape. Younger animals without a territory may select any prey they encounter, including smaller animals, because they lack the mental maps to know where to look for preferred prey of larger size (e.g., puma dispersers in Elbroch, Feltner, & Quigley, 2017a). Future work could focus on differentiating these two hypotheses.

## AUTHOR CONTRIBUTIONS

**Logan Bates‐Mundell:** Conceptualization (lead); data curation (equal); formal analysis (lead); investigation (lead); methodology (lead); software (lead); validation (lead); visualization (lead); writing – original draft (lead). **L. Mark Elbroch:** Conceptualization (equal); data curation (supporting); investigation (supporting); methodology (supporting); project administration (lead); supervision (lead); validation (supporting); visualization (supporting); writing – review and editing (supporting). **Sara H. Williams:** Formal analysis (supporting); investigation (supporting); writing – review and editing (supporting). **Kim Sager‐Fradkin:** Data curation (supporting); project administration (supporting); writing – review and editing (supporting). **Heiko U. Wittmer:** Data curation (supporting); writing – review and editing (equal). **Maximilian L. Allen:** Data curation (supporting); writing – review and editing (supporting). **Bogdan Cristescu:** Data curation (supporting); writing – review and editing (supporting). **Christopher C. Wilmers:** Data curation (supporting); writing – review and editing (supporting).

## CONFLICT OF INTEREST STATEMENT

The authors declare that they have no conflict of interest.

## Supporting information


Data S1:

## Data Availability

The datasets used and/or analyzed during this study are available in Dryad at https://doi.org/10.5061/dryad.3r2280gpv and are provided as supplementary material for reviewers.

## Appendix

**TABLE A1:** Prey weights for ungulates across all six study sites.

Ungulate type	Weight (kg)	References
Black‐tailed deer (AF)	57.38	Parker et al. (1993), WDFW, & Mammals of the PNW
Black‐tailed deer (AM)	86	Parker et al. (1993), WDFW, & Mammals of the PNW
Black‐tailed deer (AU)	71.69	Parker et al. (1993), WDFW, & Mammals of the PNW
Black‐tailed deer (SF)	51.37	Parker et al. (1993), WDFW, & Mammals of the PNW
Black‐tailed deer (SM)	65.68	Parker et al. (1993), WDFW, & Mammals of the PNW
Black‐tailed deer (yearling)	45.36	Parker et al. (1993), WDFW, & Mammals of the PNW
Chulengo (1 year)	42	Sarno and Franklin (1999)
Chulengo (2 year)	100	Sarno and Franklin (1999)
Cow	306	UC Davis (2004)
Feral horse	420	Knopff et al. (2010)
Guanaco (>2 year)	120	Raedeke (1979)
Huemul (juvenile)	5	Flueck and Smith‐Flueck (2005)
Huemul (adult)	65	Iriarte (2008)
Mule deer (AF)	75	Clark et al. (2014)
Mule deer (AM)	60	Siskiyou Project
Mule deer (AU)	68	Clark et al. (2014)
Mule deer (SM)	50	Clark et al. (2014)
Mule deer (yearling)	44	Clark et al. (2014)
Pronghorn	47	Silva and Downing (1995)
Roosevelt elk (AF)	227.59	Cook et al. (2013), WDFW, & Mammals of the PNW
Roosevelt elk (SF)	172.98	Cook et al. (2013), WDFW, & Mammals of the PNW
Roosevelt elk (SM)	285.08	Cook et al. (2013), WDFW, & Mammals of the PNW
Roosevelt elk (yearling)	118.36	Cook et al. (2013), WDFW, & Mammals of the PNW
Rocky mountain elk (AF)	315	Clark et al. (2014)
Rocky mountain elk (AM)	217	Clark et al. (2014)
Rocky mountain elk(AU)	266	Clark et al. (2014)
Rocky mountain elk (SM)	179	Clark et al. (2014)
Rocky mountain elk (yearling)	138	Clark et al. (2014)

Abbreviations: AF, adult female; AM, adult male; AU, adult of unknown sex; SF, subadult female; SM, subadult male.

**TABLE A2:** Prey weights and references for all non‐ungulate species found at kill sites.

Prey type	Weight estimate (kg)	Prey References
American robin	0.077	ADW (2020)
Badger	8	ADW (2020)
Bird	0.126	Averaged all birds <1 kg
Black bear	12	Pokrovskaya (2015)
Beaver (American)	22.5	ADW (2020) & Adirondack Ecological Center
Bobcat	9.5	ADW (2020) & Avg of 6 females captured by Makah tribe
California ground squirrel	0.509	ADW (2020)
Canada goose	6.95	ADW (2020)
Coyote	14	ADW (2020)
Culpeo foxes	9	Iriarte (2008)
Deer mouse	0.017	ADW (2020)
Douglas squirrel	0.25	animaldiveristy.org
European hares	4	Elbroch and Wittmer (2013)
Fisher	5	Patti Happe & Olympic National Park
Gray fox	5.5	ADW (2020)
Gray jay	0.072	University of Michigan Museum of Zoology
Grouse (sooty)	1.135	ADW (2020)
Grouse (ruffed)	1.14	Based on range for Grouse (blue)
Jackrabbit (black‐tailed)	2.2	ADW (2020)
Magpie (black‐billed)	0.1775	ADW (2020)
Mountain beaver	0.85	Lovejoy and Black (1974)
Muskrat	1.1358	ADW (2020)
Northern flicker	0.17	ADW (2020)
Nutria	15	WDFW
Patagonian hairy armadillo	2	Iriarte (2008)
Porcupine	9.5	ADW (2020)
Racoon	7	ADW (2020)
River otter	9.08	Avg. of 10 river otters caught by LEKT between 2012 and 2015
Snowshoe hare	1.4	Canadian Wildlife Federation
Spotted skunk	0.4665	ADW (2020)
Steller's jay	0.12	ADW (2020)
Turkey	7.3	ADW (2020)
Turkey vulture	1.425	ADW (2020)
Upland geese	6.4	Todd (1996)
Unknown mammal	6.6137	Knopff et al. (2010), Clark et al. (2014)
Varied thrush	0.0825	ADW (2020)
Western gray squirrel	0.65	ADW (2020)
Scrub jay (western)	0.085	ADW (2020)
Woodpecker	0.17	ADW (2020)

**TABLE A3:** Table of coefficients including three top models, all associated model variables, and statistical significance. Significance codes: ****p* < .001, ***p* < .01, **p* < .05.

	Estimate	Std. error	*t* value	Pr(>|z|)	Significance
Model 1					
(Intercept)	3.30546	0.119994	27.547	<2e‐16	***
Age	−0.007324	0.050343	−0.145	.8843	
Sex	0.104892	0.099089	1.059	.2898	
Season_Spring	0.381341	0.05046	7.557	4.11E‐14	***
Season_Summer	−0.078621	0.046976	−1.674	.0942	
Season_Winter	0.415553	0.052662	7.891	3.00E‐15	***
Max_preyelk	0.597859	0.124776	4.791	1.66E‐06	***
Max_preyguanaco	0.303699	0.181512	1.673	9.43E‐02	
Age:sex	0.207273	0.090223	2.297	.0216	*
Model 2					
(Intercept)	3.3598	0.1172	28.667	<2e‐16	***
Season_Spring	0.3706	0.05032	7.365	1.77E‐13	***
Season_Summer	−0.08588	0.04688	−1.832	.067	
Season_Winter	0.4106	0.05261	7.804	6.00E‐15	***
Max_preyelk	0.54471	0.12807	4.253	2.11E‐05	***
Max_preyguanaco	0.31859	0.19098	1.668	.0953	
Model 3					
(Intercept)	3.37515	0.11348	29.743	<2e‐16	***
Age	0.05228	0.04287	1.219	.2227	
Season_Spring	0.37563	0.05046	7.445	9.72E‐14	***
Season_Summer	−0.0826	0.04696	−1.759	.0786	
Season_Winter	0.41423	0.0527	7.86	3.83E‐15	***
Max_preyelk	0.5468	0.1229	4.449	8.62E‐06	***
Max_preyguanaco	0.31802	0.18286	1.739	8.20E‐02	
